# Cellulose Nanocrystals/Graphene Hybrids—A Promising New Class of Materials for Advanced Applications

**DOI:** 10.3390/nano10081523

**Published:** 2020-08-04

**Authors:** Djalal Trache, Vijay Kumar Thakur, Rabah Boukherroub

**Affiliations:** 1 Energetic Materials Laboratory, Teaching and Research Unit of Energetic Processes, Ecole Militaire Polytechnique, BP 17, Bordj El-Bahri, 16046 Algiers, Algeria; 2 Biorefining and Advanced Materials Research Center, Scotland’s Rural College (SRUC), Kings Buildings, Edinburgh EH9 3JG, UK; Vijay.kumar@sruc.ac.uk; 3 Department of Mechanical Engineering, School of Engineering, Shiv Nadar University, Greater Noida, Uttar Pradesh 201314, India; 4 Institut d’Electronique, de Microélectronique et de Nanotechnologie (IEMN-UMR CNRS 8520), University Lille, CNRS, Centrale Lille, University Polytechnique Hauts-de-France, UMR 8520—IEMN, F-59000 Lille, France; rabah.boukherroub@univ-lille.fr

**Keywords:** cellulose nanocrystals, graphene, hybrids, applications

## Abstract

With the growth of global fossil-based resource consumption and the environmental concern, there is an urgent need to develop sustainable and environmentally friendly materials, which exhibit promising properties and could maintain an acceptable level of performance to substitute the petroleum-based ones. As elite nanomaterials, cellulose nanocrystals (CNC) derived from natural renewable resources, exhibit excellent physicochemical properties, biodegradability and biocompatibility and have attracted tremendous interest nowadays. Their combination with other nanomaterials such as graphene-based materials (GNM) has been revealed to be useful and generated new hybrid materials with fascinating physicochemical characteristics and performances. In this context, the review presented herein describes the quickly growing field of a new emerging generation of CNC/GNM hybrids, with a focus on strategies for their preparation and most relevant achievements. These hybrids showed great promise in a wide range of applications such as separation, energy storage, electronic, optic, biomedical, catalysis and food packaging. Some basic concepts and general background on the preparation of CNC and GNM as well as their key features are provided ahead.

## 1. Introduction

The excessive consumption of fossil-based resources and resulting environmental problems issues coupled with the constancy growing global population requests the improvement of living standard and accelerating technology development. It has stimulated and attracted researchers worldwide to develop a sustainable bio-based alternative that can compete in performance with petroleum-based products expected to be employed in a wide range of applications [1,2,3,4,5,6,7]. Cellulose, as the most abundant bio-based material from the biosphere, has attracted more and more attention in different fields and could serve as a prominent alternative to the exhaustible fossil resources, owing to its renewability, biodegradability, biocompatibility, non-toxicity and environmental friendliness [8,9,10,11,12]. The advantages of cellulose can be also pushed forward through the exploration of its nonmetric size, which generates nanocellulose (NC), considered as a promising class for future materials due to its outstanding physicochemical properties [13,14,15,16]. NC displays low density, specific barrier properties and low thermal expansion coefficient, high strength, excellent stiffness, elongation morphology, inertness, large surface area and aspect ratio, abundance and ease of bio-conjugation [11,17,18,19]. The presence of several reactive chemical groups on its surface allows it to be modified by physical adsorption, covalent bonding or surface grafting to further extend its performance [20]. Research activities concerning NC had attracted growing interest over the past decade as reflected by the rapid increase of scientific publications and patents granted internationally [21]. According to Markets and Markets, the NC market is forecasted to achieve USD 783 Million by 2025 [13] and thus, NC production will have a high economic impact [22]. Moreover, an interesting review has been recently published by Charreau et al. dealing with the analysis of the evolution of patents involving nanocellulose since 2010 [23], demonstrating the increasing industrial interest in the this, which enabled the setting-up of the first facilities producing commercial quantities of NC.

Numerous nanocellulose types with outstanding features can be produced from different cellulosic sources employing various approaches [17,24,25,26]. NC can be divided into two main categories, that is, nanostructured materials (cellulose microfibrils and microcrystalline cellulose) and nanofibers (cellulose nanocrystals, cellulose nanofibrils and bacterial cellulose) [13]. Due to their excellent inherent characteristics, cellulose nanocrystals (CNC), as a subclass of NC, is commonly produced from cellulosic fibers and fibrils after the elimination of the amorphous regions by acid hydrolysis [23,27,28]. CNC have aroused wide scientific and technological interest from both academicians and industrials and can be utilized as an independent functional material, template support, stabilizer, filler or reinforcing agent [29,30,31]. CNC-based nanomaterials have been extensively investigated due to their unique physicochemical, mechanical, thermal, rheological and optical features. CNC could confer excellent properties to hybrids or nanocomposites (metallic, ceramics and polymeric) even at low concentration for different applications such as medical, pharmaceutics, catalysis, oil/water separation, decontamination, flame retardancy, electronic and optical devices, energy storage, sportswear, light weight armor systems, food packaging, to cite a few [10,11,13,14,20,32,33,34,35,36,37,38,39].

The combination of CNC and nanocarbons, such as fullerenes, nanotubes (single-walled, double-walled, few-walled or multi-walled), nanodiamonds and graphene-based materials (graphene, graphene oxide, reduced graphene oxide, graphene quantum dots), has recently emerged as a new class of hybrid materials for which a synergetic effect has been revealed in a wide range of applications, spanning from sensing and biosensing to catalysis, photonics and optics, energy and environment, water treatment, medical and optoelectronics. Other nanocarbons such as carbon black, activated carbon, carbon quantum dots and carbon nanofibers are less frequently used as CN-based hybrids [12,20,40,41,42,43]. 

Graphene-based nanomaterials (GNM), which have been considered as emerging and high efficient two-dimensional (2D) nanomaterials, play a crucial role in various research area since the discovery of graphene in 2004 [44,45]. They find applications in several fields such as thin-film transistors, ultra-sensitive chemical sensors and transparent conductive films, biomedical, microelectronics, composites, among others [46,47,48,49]. Recent investigations by Yang et al. provided general reviews of the whole graphene patenting activities and especially focused on the study of sustainable competitive advantages in the biomedical field [50,51]. A comprehensive review dealing with graphene, its related materials and properties have also been published [52]. Although the present development of industrial-scale graphene is still widely at the Research and Development (R&D) stage, the global graphene market reached ca. USD 78.7 million in 2019, with the request in nanocomposites, energy storage materials and semiconductor electronics, which are also underpinning future growth rate estimates of >30% per year and expected to reach >USD 221.4 million by 2025 [53,54]. Nowadays, graphene oxide (GO) materials account for >30% of the global graphene market share as progresses in GO, permitting for numerous of possibly scalable approaches to reach mass production of chemically modified graphene with a wide range of applications [54,55]. However, despite that many technically feasible approaches are currently being developed to produce efficient GNM, there are still numerous practical obstacles that need to be overcome. For instance, GNM are more frequently produced from aqueous dispersions but can easily aggregate. Such agglomeration behavior can reduce the surface area and negatively impact the mechanical, electrical and optical properties. Therefore, the incorporation of CNC not only surpasses such drawback through its excellent dispersive features but also confers further benefits to the produced GNM/CNC hybrids such as flexibility, stretchability, in addition to the improvement of the adsorption capacity, photothermal activity, stability, intrinsic luminescence and fluorescence, optical transparency and thermal conductivity [36,40,42,56].

Owing to the benefits of CNC and GNM materials as well as the numerous research works published during the last few years worldwide, a timely update on recent advancements in the field of CNC/GNM hybrid-based materials is an urgent need for both academic and industrial scientists. In this overview, we thoroughly review the recent progress made in the preparation, modification, properties and current applications of CNC/GNM hybrids in various fields. This work highlights a comprehensive overview with a forward-looking approach on CNC/GNM hybrids for numerous utilizations, which have emerged in the past five years. For the reader’s comfort and to maintain lucidity, first, some of the basic concepts dealing with CNC and GNM, their preparation and features to further elucidate their unique attributes, are discussed in brief. We will then focus on state-of-the-art cellulose nanocrystals-graphene based materials, which have mainly emerged since 2015. Few articles before 2015 are succinctly summarized in some sections.

## 2. Cellulose Nanocrystals (CNC)

### 2.1. Fundamental of Nanocellulose

**Figure 1:**
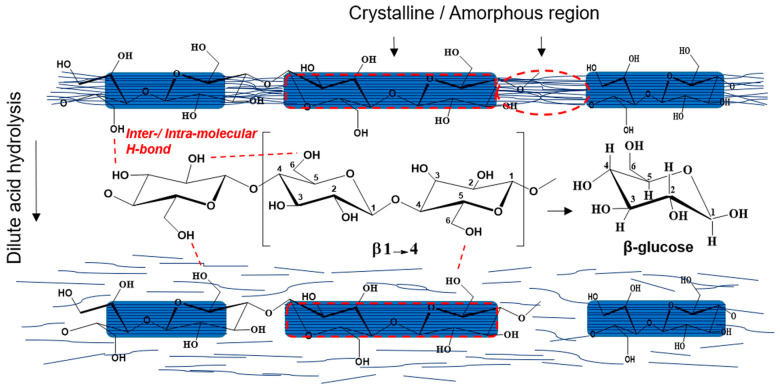
Hypothetical schematic of dilute acid pretreatment process to extract the crystalline regions of cellulose from amorphous domains. In the middle, the configuration of cellulose repeating unit with the β-(1,4) glycosidic linkage under the effect of intra/intermolecular hydrogen bonding is denoted. Reproduced with permission from Reference [36]. Copyright ©2019, Elsevier.

Cellulose, which was first extracted from wood by Enselme Payen in 1838, is a polysaccharide consisting of β-1,4-linked anhydroglucopyranoside units, in which every monomer unit is corkscrewed at 180° compared to its neighbors [5,57]. The annual production of this abundant biopolymer is estimated to be between 10^10^ and 10^11^ tons-per-year. It can be isolated from different sources such as wood, herbaceous plants, grass, crops and their byproducts, bacterial, algae and animal sources, among others [58,59,60,61]. The properties of this biomacromolecule are closely related to the natural source, its maturity and origin, pretreatment methods, processing approaches and reaction conditions. Typically, lignocellulosic biomass necessitates the removing of non-cellulosic constituents such as extractives, lignin and hemicellulose [62]. Trache et al. have recently reviewed the common pretreatment and processing methodologies, which allow the obtaining of pure cellulose from lignocellulosic [13]. As depicted in Figure 1, cellulose is semi-crystalline in nature, hence it contains both crystalline and amorphous regions. This latter is susceptible to hydrolysis; it can steadily be eliminated to generate crystalline parts upon phase segregation and is more prone to react with other molecular groups [57,63,64]. The intra- and intermolecular chemical groups (Figure 1) confer to this fascinating polymer its specific features such as infusibility, chirality, hydrophilicity, insolubility in several aqueous media and ease of chemical functionalization [65]. Cellulose can be classified into various polymorphs, that is, cellulose I, II, III_I_, III_II_, IV_I_ and IV_II_, which can be converted from one form to another through chemical or thermal treatments [42].

In their nano-size form, cellulose nanomaterials, also known as nanocellulose (NC), display outstanding physical, chemical, biological, magnetic, electrical and optical characteristics compared to the bulk materials [1,11,66]. Conceptually, NC can be produced by a top-down hydrolysis methodology through different steps, that is, (i) pretreatment processes of lignocellulosic biomass employing physical approaches concerning crushing, screening, washing and cooking to eliminate coarse particles, oily content and dust from the material surface, (ii) removing extractive/hemicellulose/lignin via chemical, physical, physicochemical, biological or the combination of two or more treatments, (iii) fragmentation and cleavage of cellulosic elementary fibrils or micro-fibrils to generate nanofibers through various approaches and (iv) post-treatments such as solvent removing, dialysis, sonication, centrifugation, surface modification, stabilization and drying. The three latter steps have received great interest from the scientific community for designing products with desired features [12,13,23,27,38,67,68,69,70,71,72,73]. NC possesses excellent useful properties such as renewability, eco-friendliness, biocompatibility, non-toxicity, hydrogen-bonding capacity, tunable crystallinity, high chemical resistance, tailored aspect ratios (100–150), low thermal expansion coefficient, reactive surface, low density (1.6 g/cm^3^), high specific surface area (100–200 of m^2^/g), high tensile strength (7.5–7.7 GPa) and elastic modulus (130–150 GPa) [10,30]. This promising polysaccharide has received tremendous attention during the last two decades in a wide range of applications such as sensors and biosensors, energy storage systems, oil and gas drilling and cementing, papermaking, filtration, decontamination, adsorption, separation, wood adhesives, Pickering emulsifiers, medical and nanocomposites, to cite a few [13,14,16,20,35,36,42,74,75,76]. Depending on the isolation method, morphology and size, NC is principally categorized into: (i) cellulose nanostructured materials such as cellulose microfibrils and microcrystalline cellulose and (ii) cellulose nano-objects, also known as nanofibers, such as cellulose nanocrystals (CNC), cellulose nanofibrils (CNF) and bacterial nanocellulose (BC).

In contrast to nanostructured materials, nanofibers present more uniform particle size distribution, high specific surface area, amphiphilic nature, barrier properties, high crystallinity and tend to produce more stable self-assembled structures such as hydrogels and films [1,30,77]. In recent years, other types of nanofibers appeared such as amorphous nanocellulose, cellulose nanoyarn and cellulose nanoplatelets [17]. Several in-depth reviews with detailed discussions dealing with NC-based materials have been reported over the past few years, covering the nanocellulose sources, isolation methods, structure modification, potential uses, advantages and shortcomings [11,16,20,23,35,36,38,40,41,78,79,80,81,82,83]. In the following, we concisely go through current extraction methods of CNC and describe their outstanding features.

### 2.2. Extraction and Properties of CNC

CNC can be often obtained from different types of lignocellulose through a top-down hydrolysis approach by combining various procedures [9,17,23,84,85]. To extract pure cellulose (PC) through the elimination of extractives, lignins and hemicelluloses, some pretreatments (chemical, physical, physicochemical, biological or their combination) of the natural source are usually required [13,34,86]. Specific treatments can be then applied to PC to produce CNC through the removing of disordered regions from pristine cellulose. The crystalline domains remain intact because of their higher resistance to the hydrolytic action, whereas the amorphous parts dispersed as chain dislocations on segments along the cellulose fibrils are more susceptible to the hydrolysis process [19,78,82]. Afterwards, the elementary fibrils are transversely cleaved, producing short CNC with somewhat high crystallinity. Nonetheless, after this process, extra post-treatments such as solvent removing, sonication, fractionation, dialysis, centrifugation, filtration, washing, stabilization, surface modification, neutralization and drying are required to recover CNC product.

**Figure 2:**
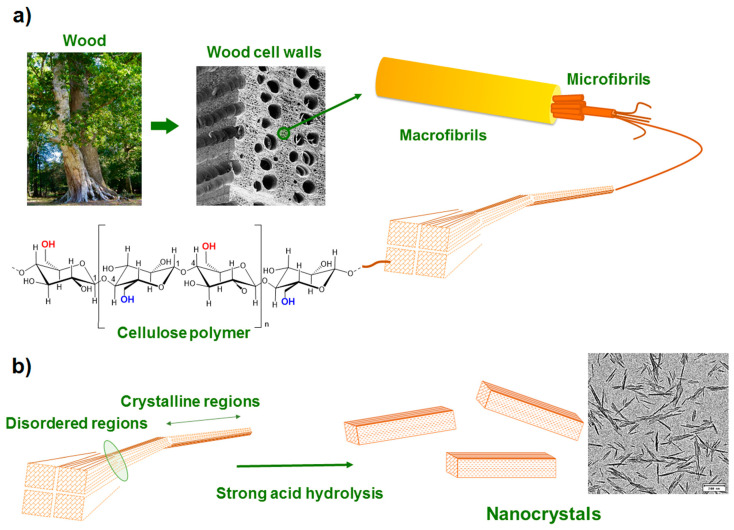
(**a**) Structural hierarchy of the cellulose fiber component from the tree to the anhydroglucose molecule. (**b**) Preparation of cellulose nanocrystals (CNC) by selective acid hydrolysis of cellulose microfibrils. Reproduced with permission from ref [99]. Licensed under a Creative Commons Attribution 3.0 International License (https://creativecommons.org/licenses/by/3.0/).

The most common hydrolysis method used to produce CNC relies on sulfuric acid, which can react with the surface hydroxyl groups of pristine cellulose through an esterification process, allowing the grafting of anionic ester groups [77,87]. This latter generates a negative electrostatic layer that covers nanocrystals, promoting the dispersion of CNC in water but reducing their thermal stability. Recently, as an alternative to sulfuric acid hydrolysis, other liquid inorganic acids such as nitric, hydrobromic, phosphoric and hydrochloric have been extensively reported [11,30]. The preparation of CNC from wood, for which the hydrolysis process causes preferential digestion of the amorphous part of cellulose while the ordered regions remain intact, is schematized in Figure 2. Both natural source and experimental conditions (acid concentration, reaction time, temperature, mass ratio, etc.) may influence the characteristic of the prepared CNC such as crystallinity, dimensional dispersity, thermal behavior, mechanical properties, density, aspect ratio and morphology. Although the hydrolysis process using mineral acids is simple and not time-consuming, certain drawbacks such as lower yield, high amount of water usage, severe environmental pollution and harsh corrosion of equipment should be overcome [30]. Therefore, to address the above issues, various recent procedures such as organic acid (oxalic, formic, etc.) hydrolysis [88], solid acid (phosphotungstic) [89], subcritical water hydrolysis [90], deep eutectic solvents [91], ionic liquids [92], oxidation [93], sonication [94], enzymatic [95] and combined approaches [5,17,31] have been applied and others continue to be developed worldwide to produce CNC with desired properties at lower costs and higher yield based on sustainability principle and environmentally friendly policy [5,13,17,31]. Nevertheless, scaling-up from laboratory to industrial scale remains one of the most important issues and considerable efforts should be made to prevail over the remaining constraints. Otherwise, some companies such as CelluForce and Alberta Innovates, among others, produce CNC at large scale [13,96].

CNC present unique features compared to the other classes of NC with the spotlight to characteristics such as physical, chemical, optical, thermal, mechanical, electrical properties [1]. CNC consist of an elongated, needle or rod-like nanoparticles. They are 4–70 nm in width and 100–6000 nm in length and aspect ratio of 5–70, as well as large surface area (150–500 m^2^·g^−1^), which allows it to be easily dispersed in water to generate a chiral nematic organization [13,16,97]. CNC also exhibit high crystallinity (50%–90%), a tensile strength of up to 7.5 GPa, a Young’s modulus of ~170 GPa and a bending strength of about 10 GPa [9,13,68]. They also display good thermal stability up to 200 °C and can find applications in processes like thermoplastics [97]. Nevertheless, these features depend closely on the source of feedstock, extraction methods and experimental conditions, which will ultimately define their applicability [98].

It is worthy to note that the abundance of –OH or other reactive chemical groups and the high surface area to volume ratio render CNC highly reactive and easy to be functionalized [100]. Therefore, to improve their compatibility and ensure a good dispersion, CNC surface can be chemically, physically or enzymatically modified to impart stable negative or positive electrostatic charges on their surface [13,101]. Such modifications may allow tailoring the properties of the CNC-based materials depending on the intended application.

## 3. Graphene-Based Nanomaterials

### 3.1. Nomenclature and Fundamental Aspects

Graphene, discovered by Geim and Novoselov in 2004, is relatively a new two dimensional (2D) sheet-like material in which a honeycomb or hexagonal structure with a flat lattice configuration, completely composed of sp^2^ hybridized carbon atoms that are covalently bound, is densely packed [102,103]. Graphene, an atomic layer of graphite, is the unique carbon’s allotrope, where each atom is tightly linked to its neighbors by an only electronic cloud in which a C–C bond distance is 0.142 nm [104]. It is considered as a fundamental basis for all carbon allotropes and as the mother of a graphitic family for all the dimensions.

**Figure 3:**
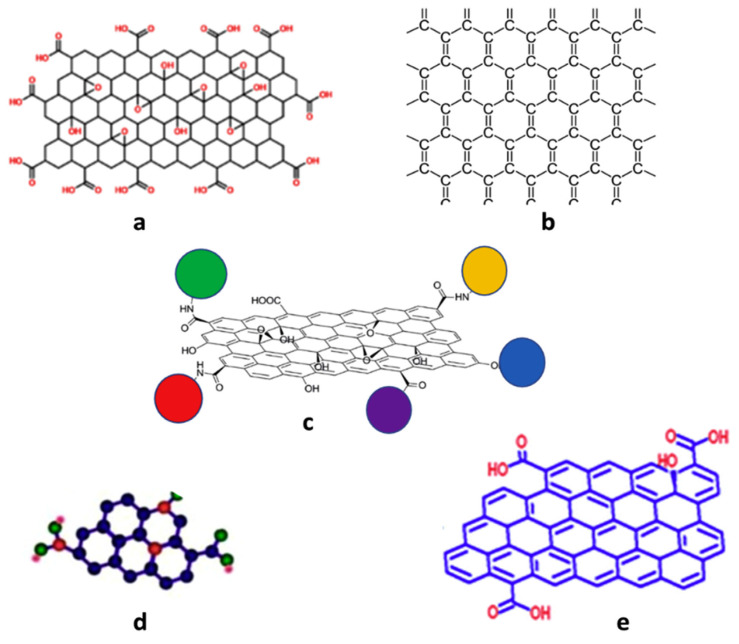

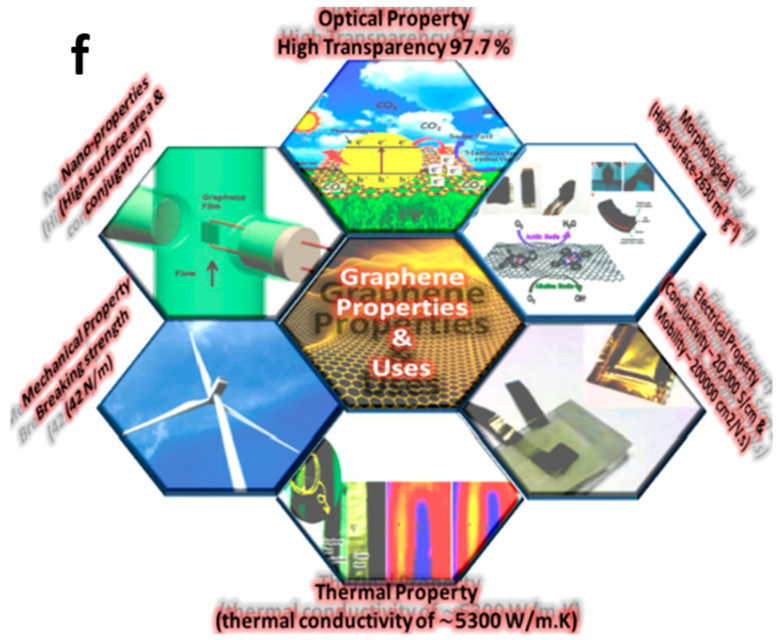
Some common forms of graphene: (**a**) graphene oxide, (**b**) pristine graphene, (**c**) functionalized graphene, (**d**) graphene quantum dot and (**e**) reduced graphene oxide. Reproduced with permission from Reference [46]. Licensed under a Creative Commons Attribution 3.0 International License (https://creativecommons.org/licenses/by/3.0/); (**f**) Different properties of graphene and its applications. Reproduced with permission from Reference [108]. Copyright ©2019, Elsevier.

Graphene-based nanomaterials (GNMs), the first materials reported as examples of 2D nanocarbons, can be classified based on the number of sheet layers, surface modifications, total oxygen content or orientation [105]. Graphene is highly hydrophobic and is prone to agglomeration, owing to the strong van der Waals’ interactions between the 2D graphene sheets, leading to low surface area and ineffective use of its outstanding features [106]. These latter are also closely dependent on the graphene availability as a single layer because if the layers are in close vicinity to each other, they are likely to restack or agglomerate due to π–π interactions. Hence, its functionalization is commonly required to surpass these issues. Typically, three types of functionalization approaches through covalent (nucleophilic substitution, electrophilic substitution, condensation and addition), noncovalent (π–π bonding, electrostatic attraction and hydrogen bonding, etc.) or a combination of both interactions can be used, where the aromaticity of graphene can be either lost or preserved [107]. As shown in Figure 3a–e, GNMs can be found in various forms for which the most important ones that will be the focus of the present review are graphene nanosheets (GNS), graphene nanoplatelets (GNP), graphene oxide (GO), reduced graphene oxide (RGO) and graphene and graphene oxide quantum dots (GQD). In the frame of the present review, the acronym GN will encompass GNS and GNP.

Graphene oxide (GO), commonly prepared from the oxidation of graphite, consists of a few- or a single-layer sheet. GO sheets are rich in various oxygen-containing groups such as hydroxyl, epoxy, carboxyl, carbonyl, phenol, lactone and quinone, which can change the van der Waals interactions. The two former chemical groups are mostly present on the basal plane, whereas the others with small quantities are found at the sheet edges. These functional groups in GO can deeply influence its electrochemical, mechanical and electronic features. Despite the aromaticity of graphene is lost in GO, owing to exploitation of π electrons in the covalent bonding of these oxy groups on graphene backbone, the carbonyl, carboxyl and so forth groups at the edge render them more dispersible in both organic solvents and water [44,107]. The hydrophobic aromatic frameworks and the hydrophilic oxygen-containing groups make GO amphiphilic, allowing its interaction with inorganic and organic molecules.

Reduced graphene oxide (RGO), obtained by the reduction of GO [109], contains fewer oxygen atoms, hence, is less negatively charged [106]. During the reduction, RGO recovers the graphitic arrangements (partial recuperation of the sp^2^ from sp^3^ hybridization of GO) through the elimination of the oxygen-containing groups, which have been inserted in the oxidation step, thus, restoring the electronic properties of graphene [110]. This partial reduction and the exposure to some chemicals allow tailoring the conductivity, band-gap and optical features of the material [111,112].

Graphene quantum dots (GQDs), which can be found as single- or multiple layers, display interesting features such as good chemical stability, high surface area, tunable physical characteristics, stable photoluminescence and low toxicity [113,114]. They can be used in optoelectronic, electronic, biomedical, sensors and energy storage. They usually consist of up to 10 layers of 10–60 nm size RGO [46].

Graphene-based nanomaterials possess exceptional electrical, optical, mechanical, electrochemical and thermal features that make them versatile for a wide range of applications and have drawn worldwide attention in both academic and engineering fields [44,105]. They can be employed in industrial applications such as biomedical, solar cells, biosensors, supercapacitors, electromagnetic absorbers, optical devices, integrated circuit, protective coatings, organic light-emitting diodes, sound transducers, petroleum industry, automobile components, aerospace, energy storage, nanocomposites and contamination purification in wastewater management, to cite a few (Figure 3f) [107,115,116].

### 3.2. Synthesis Routes and Properties

**Figure 4:**
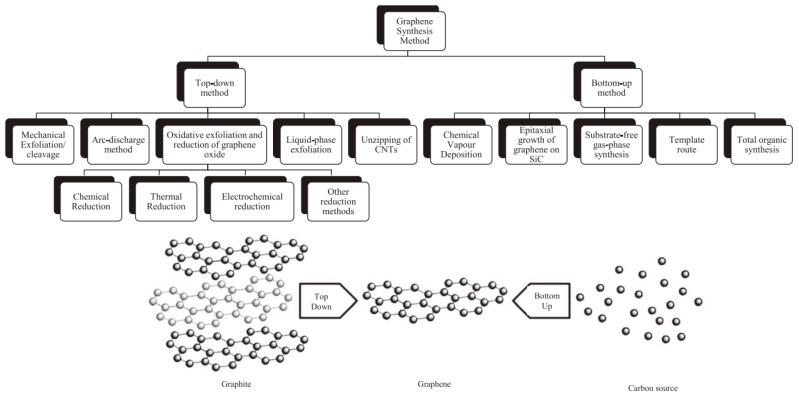
Production techniques of graphene materials. Reproduced with permission from Reference [119]. Licensed under a Creative Commons Attribution 3.0 International License (https://creativecommons.org/licenses/by/3.0/).

Graphene can be produced from various sources such as graphitic, non-graphitic and waste materials using top-down or bottom-up approaches [117,118,119,120]. The common routes for its fabrication are summarized in Figure 4. The top-down synthesis routes encompass mechanical exfoliation, liquid phase-exfoliation (LPE), oxidative exfoliation-reduction, arc discharge, unzipping of carbon nanotubes, for which larger precursors such as carbon-based materials or graphite are destroyed to produce a single-, bi- and few-layer graphene. Broadly, some of these approaches can generate high-quality products and are likely scalable. Nevertheless, they provide limited yield and have complications in making nanomaterials with reliable characteristics, which are closely dependent on the carbon precursor. On the other hand, the bottom-up synthetic routes could produce graphene using atomic-sized precursors. These approaches comprise epitaxial growth, chemical vapor deposition (CVD), total organic synthesis, template route and substrate-free gas-phase synthesis. Despite the quality of the produced graphene is better than that generated using top-down methods, they often require advanced operational setup, high fabrication costs and are energy-consuming. Further advantages and the shortcomings of the most important methods have been reported elsewhere [117,118].

It is worthy to note that most of the studies have not usually utilized graphene in its pristine form, because of its lower yield from the production point of view. Therefore, its derivatives have received much attention. GO is commonly prepared using a chemical oxidation process of graphite with subsequent dispersion and exfoliation in a suitable solvent (e.g., water). Graphene oxide sheets can also be fabricated using a modified Hummers’ method, which is described in several reports [121,122]. The oxidation processes can lead to fragmentation, crack, winkle, structure disorder, impurities and defects that may influence the adsorption, optical and electronic characteristics of GO. RGO, however, is usually produced by reducing graphene oxide employing different ways such as chemical, thermal, photocatalytic and electrochemical reductions [123]. Nonetheless, the obtained RGO may contain some impurities with the presence of structural defects. Besides that, the production strategies of GQDs comprise solvothermal, microwave, CVD and soft template processes, in-situ reduction of GO, electrochemical fabrication, chemical synthesis and electron beam lithography [113,114,124]. Among them, top-down approaches have been proved to be the most appropriate and cost-effective methods [46]. GQDs exhibit similar features compared to various types of quantum dots (QDs), particularly in the case of inorganic QDs [113].

It has been recently revealed that oxidative exfoliation-reduction, liquid-phase exfoliation and CVD are the most interesting production methods, which possess high potential for industrial implementation to produce graphene-based nanomaterials [45]. However, to develop effective synthesis processes of graphene and its derivatives, further research activities have to be conducted to improve the quality, yield of the products with tailorable properties using cost-effective, environmentally friendly, reliable and scalable approaches.

The properties of graphene-based materials are closely dependent on the number of layers as well as the extent of defects. Graphene, as the thinnest carbon material, presents outstanding features such as higher surface area of ~2630 m^2^/g compared to GO and other derivatives. It has been reported that a single layer of graphene absorbs 2.3% of white light with a reflectance of less than 0.1%. At room temperature, the in-plane thermal conductivity of GN is about 2000–5000 W/m·K. Such dissimilarity is due to the dissemination of phonons pathway at the surface [46,108]. Some research works reported that the charge transporters and carriers mobility of 200,000 cm^2^/V·s can be reached at electron densities of ~2 × 10^11^ cm^−2^ [108]. GN possesses good chemical stability and quantum Hall effect at ordinary temperature, intrinsic strength of 130 GPa, Young’s modulus of 1.0 TPa, shear strength of 60 GPa and fracture stress of 97.54 GPa [123]. It is considered as one of the strongest materials ever tested (200 times than steel) [125]. More details about the characterization methods and the properties of graphene and its derivatives have been extensively reviewed in recent years [105,123,124,126,127].

## 4. Preparation, Properties and Application of CNC/GNM Hybrids

CNC has aroused a tremendous amount of interest of the scientific community in recent years due to its outstanding features and can be employed as an independent functional material, template support, stabilizer, filler or reinforcing agent. Recently, it has been combined with numerous GNMs such as GN [128], GO [129], RGO [130], GQDs [131,132,133], free-standing graphene (FSG) [134] and graphene nanoscrolls [135] to produce hybrid materials with excellent thermal, mechanical, optical and electronic properties. However, several scientific and technical issues can be encountered during the production of such hybrid materials such as agglomeration, limited dispersion, process scalability and high costs, among others. Hence, many research works have been carried out and others continue to be conducted worldwide to overcome these problems and obtaining efficient CNC/GNM for a wide range of applications. The emphasis of the following subsections will be dedicated to the most important approaches used to produce CNC/GNM hybrids as well as their properties. Specific attention will be dedicated to the investigations performed during the last few years.

### 4.1. CNC/GN

GN possesses large surface area, exceptional electrocatalytic activity, high mechanical strength, good electronic transport characteristics, excellent optical properties and thermal performance, which has motivated its broad application prospect in several fields such as functional composites, electrochemical sensors and catalysis, among others [136]. To expand the number of applications of GN and enhance its inherent properties, numerous GN composites have been successfully produced and applied in several fields. Recently, nanocomposites of CNC/GN have attracted widespread attention, owing to their exceptional features and synergetic effects that develop new ways and opportunities for the production, characterization and application of new materials in nanotechnology. It has been recently demonstrated that the preparation of CNC/GN can be carried out with and without chemical functionalization for which the water-based dispersion is the common starting approach to produce composites with/without a combination with various types of materials such as metallic and ceramic nanoparticles or natural and synthetic polymers. Several processes can be further applied such as filtration, hot pressing, deposition and drying to generate a wide range of advanced materials. Thus, such CNC/GN-based materials hold a great promise for several applications ranging from packaging to biomedical fields. Nevertheless, the optimization of the composite compositions and tailoring of their properties can extend the number of applications and reduce the production cost for eventual scalability.

**Figure 5:**
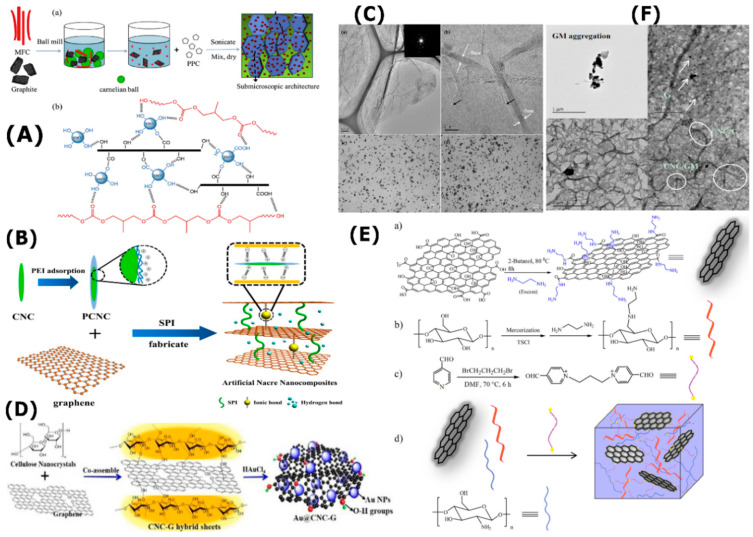
(**A**) Schematic illustration of the production of poly(propylene carbonate) (PPC)/cellulose nanocrystals (CNC)/aminated graphene (GN) composites and the available hydrogen bonding. Reproduced with permission from Reference [138]. Copyright ©2018, Elsevier; (**B**) Schematic presentation of the SPI-based nanocomposite film. Reproduced with permission from Reference [139]. Licensed under a Creative Commons Attribution 3.0 International License (https://creativecommons.org/licenses/by/3.0/); (**C**) Transmission electron microscopy (TEM) and optical micrographs of the CNC/GN solution showing good dispersion. Reproduced with permission from Reference [140]. Copyright ©2015, Elsevier; (**D**) Schematic synthesis of Au@CNC-GN catalyst. Reproduced with permission from Reference [141]. Copyright ©2018, The Royal Society of Chemistry (RSC) on behalf of the Centre National de la Recherche Scientifique (CNRS) and the RSC; (**E**) Preparation procedure of chitosan/WN/GN hydrogel. Reproduced with permission from Reference [142]. Copyright ©2020, Elsevier; (**F**) TEM images of GN and CNC/GN sol mixtures containing 2 wt.% of GN. Reproduced with permission from Reference [143]. Licensed under a Creative Commons Attribution 3.0 International License (https://creativecommons.org/licenses/by/3.0/).

Carrasco at al. have employed CNC as an effective graphene stabilizer in aqueous dispersion at high concentration for which the exfoliation of graphite to generate graphene flakes has been carried out using a tip sonication [137]. Such an approach based on CNC-assisted liquid-phase exfoliation (LPE) produced graphene flakes decorated with CNC stabilizers with interesting properties. The authors proved that such hybrids could be employed in different applications such as composites and supercapacitors. In another study by Cui et al. an interesting efficient one-step mechanical-chemical method to in-situ produce CNC/GN hybrid, with rigid 2D structure and improved interfacial interactions, from micro fibrillated cellulose and graphite using ball milling has been developed [138]. A schematic illustration of the composite preparation is given in Figure 5A. This hybrid was successfully dispersed within poly(propylene carbonate) (PPC) with strong interfacial interactions which can increase its glass transition temperature (*T*_g_) and enhance its mechanical and electrical features for practical uses. The obtained PPC/CNC/GN composite displayed a *T*_g_ of 51.3 °C, which is higher than that of pure PPC (34.0 °C). The percolation threshold considerably decreased from 15 to 5 wt.%, whereas the tensile strength and the Young’s modulus reached 52.8 MPa and 731.2 MPa, respectively.

Montes et al. have recently demonstrated the existence of a synergetic reinforcement of poly(vinyl alcohol) (PVA) nanocomposites with CNC-stabilized graphene [144]. They produced CNC/GN hybrid using a CNC-assisted LPE that allows the stabilization of the resulting GN in aqueous dispersion. Such hybrid was incorporated into a PVA aqueous solution by a direct blending to obtain a nanocomposite after casting evaporation. It was mentioned that the thermal stability of the composite is improved through the addition of 1 wt.% of CNC/GN hybrid nanofiller. Moreover, the mechanical features have been also enhanced compared to the neat PVA (20% improvement in tensile strength and 50% in Young’s modulus). It was claimed that CNC played a dual role, where it acts as GN stabilizer and PVA reinforcement. Moreover, the synergetic effect of CNC/GN hybrid is notable, where interesting thermal, mechanical and electrical features can be attained through the tailoring of the nanofiller loading. Recently, this research group has studied the effect of CCN/GN hybrid on the properties of poly(lactic acid) (PLA) based film [145]. The composite was prepared using a melt blending method, a conventional technique for plastic compounding, at a total loading of 1wt.% and then processed by hot pressing to generate the film. Compared to the baseline, the film PLA/CNC/GN exhibited high thermal stability and better mechanical features with an increase of 11 and 8% in the tensile strength and Young’s modulus, respectively. The investigation of the gas barrier properties as well as the antifungal activity of the prepared film revealed significant improvements, which make it a potential candidate in food packaging trays and agricultural film applications.

A few years ago, an interesting composite based on soy protein isolate (SBI) and CNC/GN has been developed by Li et al. as food packaging material [139]. A schematic presentation of the nanocomposite preparation is shown in Figure 5B. The authors exploited the high aspect ratio of 1D CNC with the flexible and strength 2D GN to manufacturing active interfacial adhesion laminate nanocomposites. To obtain a stable aqueous graphene dispersion via sonication, the negatively charged sulfate ester groups of CNC were firstly modified through the incorporation of positively charged surface functionalities using the cationic polyethyleneimine. Such modification enhanced the strong ionic interactions with negatively charged GN for efficient dispersion and later-by-layer assembly with SBI. The obtained composite film displayed interesting mechanical features and improved surface hydrophobicity for which the tensile strength increased from 3.75 to 7.49 MPa and the water contact angle augmented from 39° to 54° compared to the control film. Better thermal stability, water resistance and UV-visible light barrier ability were also exhibited by such composite film, making it a potential candidate as food packaging material.

Valentini et al. produced polymer solar cells using optically transparent conductive GN and CNC film [146]. They reported that the mixture containing 10 mL of CNC suspension (0.5 wt.%) and 10 mL of GN solution (1 wt.%), which was prepared in an ultrasonic bath at room temperature for 20 min and evaporated under a nitrogen stream, was the best composition. The obtained NG/CNC layer, which has a low surface roughness, was optically transparent and enabled light to go through. The measurement of the contact angle of GN/CNC demonstrated a lower contact angle value when compared to those of the neat glass or CNC film, which was assigned to the flatter surface morphology of the GN/CNC film. These authors produced a photovoltaic device by spin coating. The thickness of the spin-cast photosensitive layer was about 100 nm, as determined by atomic force microscopy. The manufactured polymer solar cell reached a higher short-circuit current density value, revealing its improved electron blocking action. The enhanced mechanical properties, the optical transparency as well as the electrical conductivity of the hybrid layer will certainly allow the development of the next generation of flexible and foldable printed optoelectronic devices. In another work, Wang et al. combined GN and CNC to produce flexible, electrically and thermally conductive hybrid thin film using a water-based approach and vacuum filtration [140]. Figure 5C shows the transmission electron microscopy (TEM) and optical micrographs of the obtained GN of about 15 layers as well as CNC/GN solution, revealing that GN was uniform without the appearance on any segregation after mixing with hydrophilic CNC. The better particle alignment with the removing of the internal pores was promoted by the use of the hot-press process. It was found that the hot-pressed 25 wt.% CNC hybrid paper exhibited interesting mechanical features for which the modulus was improved by 57% and tensile strength by 33% with respect to the neat GN paper. The electrical conductivity was negatively affected by the increase of the amount of CNC and the optimum CNC loading was 15%. Such hybrids can find application in heat and electrical-conducting fields.

The employment of CNC/GN hybrid as a supporting material to produce supported metal catalysts, which can be used as dispersing, capping or reducing agents, using a clean, simple and effective process has been reported. Wang et al. have deposited mono-dispersed gold nanoparticles (Au NPs) on multifunctional CNCN/GN hybrid sheets to generate catalysts with efficient catalytic activity, flexibility and stability [141]. The production procedure is briefly illustrated in Figure 5D. The hybrid structure allowed the reduction, growth and immobilization of Au NPs. The OH-groups of CNC coordinated with GN permitted creating narrow nanosized Au NPs anchored onto the surface of the hybrid through the in-situ reduction of Au^3+^. Such a catalyst has been revealed to be effective for one-pot reaction of an alkyne, an amine and an aldehyde in water since it can minimize the environmental pollutions caused by heavy metallic ions and organic solvents. It can be reused for several times without significant deactivation. It is also expected to be employed in a wide range of applications such as energy storage and catalysis.

On the other hand, stimuli-responsive hydrogels, such as 3D polymeric networks, are considered prominent intelligent drug delivery systems to selectively release the drug at the desired sites. The emerging of CNC/GN hybrids has pushed the scientific community to develop a new generation of efficient hydrogels. Omidi et al. have successfully developed a pH-responsive hydrogel containing aminated CNC (WN), aminated graphene (GN) and chitosan via Schiff base reaction by a synthetic dialdehyde in a few minutes [142]. The preparation procedure is schematized in Figure 5E. The prepared hydrogel exhibited better sensitivity to different external stimuli encompassing pH and amino acids. More specifically, it displayed a pH-responsive release behavior for anticancer drugs. Also, the hydrogel presented strong antibacterial activity against gram-positive bacteria, revealing the efficiency of such hydrogel as a potential candidate for the localized drug delivery systems.

To extend the application of CNC/GN hybrids as anti-static or electromagnetic interference shielding materials, Liu et al. have prepared sandwiched films of epoxy resin and GNC/GN paper. Firstly, a hybrid paper of GN containing 10 wt.% of CNC was produced using ultrasonication process in aqueous suspension [147]. This hybrid displayed an electrical conductivity of 16,800 S/m and tensile strength of 31.3 MPa. To manufacture the sandwiched film of epoxy and CNC/GN, a dip coating method was applied through the introduction of the paper into epoxy resin solution followed by a curing process at ambient temperature. The authors revealed that the moduli of the films were about 300 folds and the tensile strength increased by two-folds concerning the pure resin. The glass transition of the composite increased as well when compared to that of the neat resin. Besides, the coated CMC/GN hybrid by epoxy resin displayed better dimensionality integrity after sonication in water for two hours. In another work, a composite containing water-born polyurethane/CNC/GN has been recently prepared through one-step sol process by Yang et al. as a thermosetting coating material for wood-based composites, which exhibited better energy-saving characteristics [143]. The better dispersion of CNC/GN has been optimized as shown in a TEM image in Figure 5F. The properties of the prepared composites have been improved through the incorporation of CNC/GN, where the thermal conductivity, abrasion resistance and hardness were enhanced and meanwhile, the coating adhesion was maintained at an acceptable level. The authors claimed that such findings can promote the development of wooden heating material with better-energy saving characteristics.

**Figure 6:**
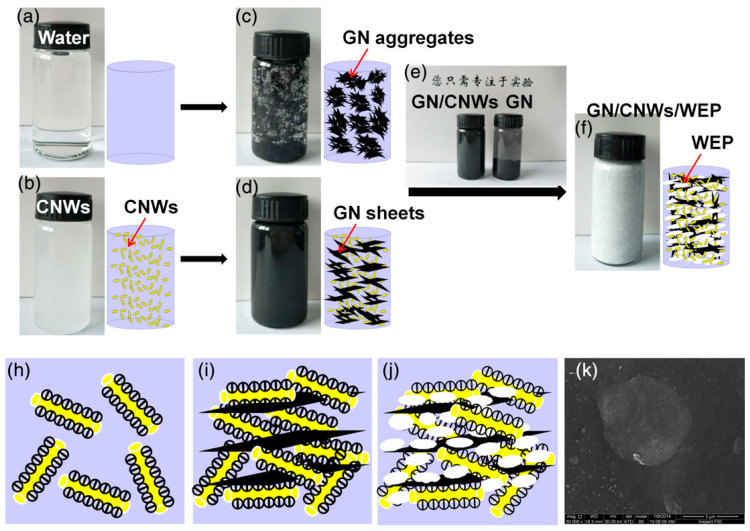
Neat water (**a**); aqueous dispersion of CNC (CNWs) (**b**); dispersion of GN in water (**c**); GN in CNWs aqueous dispersions (**d**); dispersion stability of GN in water and CNWs aqueous dispersions after the settlement of 30 min (**e**); dispersion of 1.0% GN/CNWs in waterborne epoxy polymeric matrix (WEP), (**f**); schematic of CNWs with negative charges (**h**); schematic of negatively charged CNWs adsorbed on graphene sheets (**i**); schematic of GN sheets stabilized in WEP assisted with CNWs (**j**); Field emission scanning electron microscopy (FE-SEM) micrograph of CNWs adsorbed on graphene sheet (**k**). Reproduced with permission from Reference [148]. Copyright ©2019, Wiley.

In another study, Nie et al. have introduced a small amount of CNC (also named CNWs) to GN (CNWs/GN = 1/20 *w*/*w*) to improve its uniform dispersion in a waterborne epoxy polymeric matrix (WEP), which is still challenging at a high GN loading, using a solution-casting approach [148]. A schematic illustration of the preparation of the composite is depicted in Figure 6. The obtained film at a GN loading of 1.0 wt.% achieved enhanced mechanical properties with a higher Young’s modulus of 2820 MPa compared to the neat epoxy (2034 MPa). The glass transition of such composite increased by 4.3 °C when compared to the pure resin. The better dispersion of GN on the surface of epoxy owing to the effect of CNWs led to the increase of the water contact angle, confirming the improvement of the water-barrier behavior of the composite CNWs/GN/WEP. The authors have assessed the anticorrosion effectiveness of the prepared coating composite using potentiodynamic polarization and electrochemical impedance spectroscopy tools. The results demonstrated that CNWs played a double role through improving the dispersion of GN and the corrosion resistance for mild steel.

### 4.2. CNC/GO

GO, as one of the most important derivatives of graphene, contains a high density of oxygen-functional groups, which can allow covalent, ionic or hydrogen interactions with numerous polymeric matrices, paving the way to several technological applications. It displays interesting features such as high specific surface area, high binding potential, high hydrophilicity, high dispersibility, superior mechanical properties and surface functional groups that can be employed as attachment sites [149]. However, to fully exploit the potential of GO, the production of GO-based composites is significant. Various GO-based nanocomposites have become increasingly mature in several fields. Recently, studies on CNC/GO hybrids have been actively conducted. CNC with outstanding features such as high Young’s modulus, high crystallinity, high surface chemical activity and tailorable surface characteristics can offer strong, non-toxic and flexible advanced GO-based hybrids, which further inherit the features of both CNC and GO for which desired properties can be obtained. CNC/GO-related material composites have been investigated both on their own and after incorporation of other components such as metals, ceramics or polymers to modulate their final properties for specific uses. These hybrids found applications in nano paper, food packaging, biomedical, energy storage, sensors, decontamination, catalysis, adsorption, shape memory devices, foams, fire retardants and insulating materials, to cite a few.

**Figure 7:**
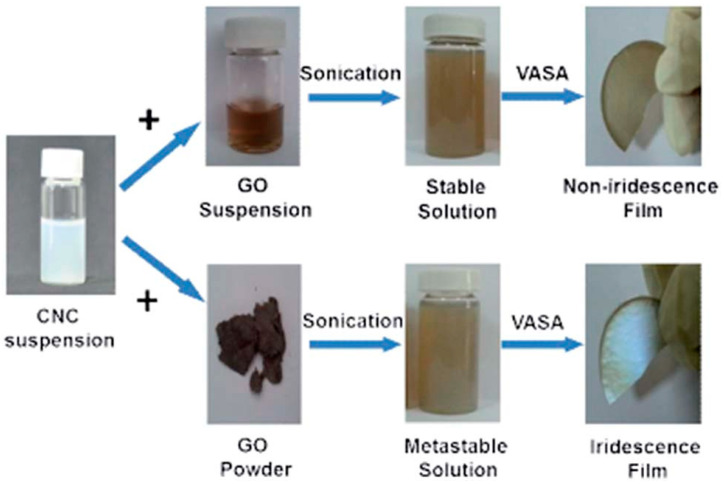
Schematic illustration of the preparation process of GO/CNC hybrid films with or without iridescence. Reproduced with permission from Reference [151]. Copyright ©2014, The Royal Society of Chemistry.

The aqueous GO solution with CNC is considered the common approach used to prepare CNC/GO composites. Kafy et al. have produced CNC/GO film as humidity sensor using this blending method, followed by the drying process [150]. They synthesized CNC using the conventional H_2_SO_4_ hydrolysis method, whereas they produced GO by way of the modified Hummer’s method. Then, they mixed the two suspensions at a desired ratio followed by homogenization. After that, the solution was poured in a petri dish and dried. The obtained hybrid exhibited a good dispersion of GO in CNC matrix for which high dielectric constant and low dielectric loss have been revealed, owing to the special polarization dipoles in CNC. These authors prepared a renewable, flexible and cheap sensor using this hybrid and an interdigital transducer patterned electrode deposited on a polyethylene terephthalate (PET) substrate. This sensor displayed a good sensitivity to humidity even under different temperatures. Similarly, Chen et al. produced CNC/GO using an aqueous suspension of CNC with either GO suspension or GO powder (Figure 7) [151]. The mixing process generated a stable solution for the first, whereas a metastable solution was obtained when GO powder was used. The drying process carried out via vacuum-assisted self-assembly technique (VASA), engendered non-iridescence and iridescence films, respectively, for the hybrids containing GO suspension and GO powder. It is worthy to note that CNC-based iridescent films found applications in optical functional materials. It was demonstrated that self-organized film was obtained from stable solution, while the separated structure was generated from the metastable solution. Interestingly, the later film, which displayed iridescent optical properties, consisted of self-assembled liquid crystals phase of CNC with embedded GO sheets. The authors claimed that such iridescent hybrid can be applied in security materials, reflective filters, sensors and other photonic materials.

Although the water-based dispersion is the widely adopted method to produce CNC/GO, it represents an unavoidable issue of the higher resistance. Valentini et al. have developed a method to produce CNC/GO with reduced electrical resistivity [129]. They employed the same approach of the mixing of the CNC suspension with the GO solution but assisted by an external electric field. This latter induced de-oxygenation of GO and hence its conductivity can be recovered to some extent. Such electrical conductivity was rather moderate, because of the presence of CNC as an insulating matrix. In a separate work of the same authors, the above approach, which is based on the drop-casting of an aqueous solution of CNC/GO between two metal electrodes, was found to be efficient to produce a resistive memory device based on CNC/GO thin hybrid [152]. Such thin film-based device exhibited a transition between low and high conductivity states upon changing the polarity of the applied external electric field. The authors claimed that such an achievement could promote the development of post-silicon electronic devices based on the integration of CNC/GN thin hybrids. Recently, Pan et al. developed a new method to produce chiral smectic structures through self-assembling 2D GO and 1D CNC nanorods [153]. Such a structure is closely dependent on the ratio of nanorods and nanosheets as well as the concentration of the composite colloid. The authors initially mixed CNC and GO suspensions at low concentration (<1%) and incorporated cross-linked polyacrylate hydrogel to concentrate the blend suspension. The CNC/GO was recovered by spin-coating of the colloid on PES substrate and dried at 60 °C. This method was considered timesaving compared to traditional approaches. It was demonstrated that such advancement can pave the way to develop optical metamaterials for optical modulation and mechanochromic sensors.

It was reported that poor dispersion of reinforcements at the nanoscale in addition to the weak interfacial interactions can negatively affect the material strength, toughness and other properties. Thus, several physical or chemical modifications can be employed to overcome such issues. In the case of CNC/GO composites, several approaches have been proposed to improve their efficiency for numerous applications. The common ones used to enhance the interfacial of such hybrids were based on the modification of CNC surface features, whereas few modifications have been simultaneously applied to CNC and GO. 

**Figure 8:**
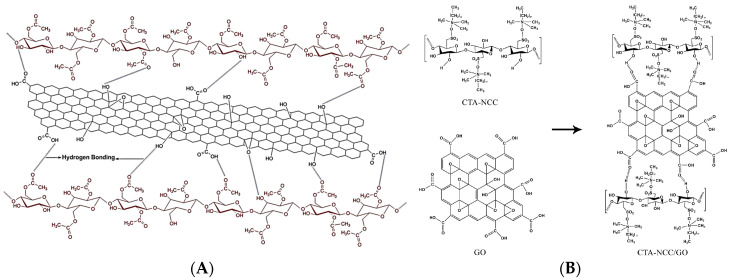
(**A**) Schematic interaction of CNCA/GO. Reproduced with permission from Reference [156]. Copyright ©2014, Springer; (**B**) Schematic diagram of CTA-NCC/GO nanocomposite. Reproduced with permission from Reference [157]. Copyright ©2019, Elsevier.

One of the interesting production methods was that developed by Xiong et al. to manufacture ultra-robust transparent CNC/GO membrane with high electrical conductivity [154]. These authors improved the interfacial interactions of anionic CNC, prepared by H_2_SO_4_ hydrolysis and anionic GO sheets obtained through the modification of CNC with 10 wt.% cationic polyethyleneimine to introduce positive surface charge functionalities. This modification enhanced the ionic interactions between the strongly positively charged polymer and negatively charged flexible GO, which consequently improved the layer-by-layer assembly, carried out on a sacrificial layer of cellulose acetate on a silicon wafer, to design laminated nanohybrids with high flexibility, outstanding mechanical strength, high optical transparency along with excellent toughness. The authors claimed that such CNC/GO hybrids could be used for a wide range of technological applications, encompassing wearable electronic devices, biofluid separation, electromagnetic interference shielding and ballistic protection. The authors also employed the same approach to produce CNC/RGO hybrids after the electrochemical reduction of the former membrane [155]. In another work, Kabiri et al. produced acetylated CNC (CNCA), which was further used to prepare well dispersed CNCA/GO hybrid by a solvent casting method (Figure 8A) [156]. It was stipulated that the modification of CNC will promote its interfacial adhesion and miscibility with GO via hydrogen bonding. It was proved that composite supplemented with 0.8M of GO offered better thermal stability, interesting mechanical properties with an increase in the tensile strength of 61.92% with respect to CNCA. Moreover, the barrier characteristics against water were improved. The authors claimed that such a composite could find potential application in electrical and electrochemical fields.

**Figure 9:**
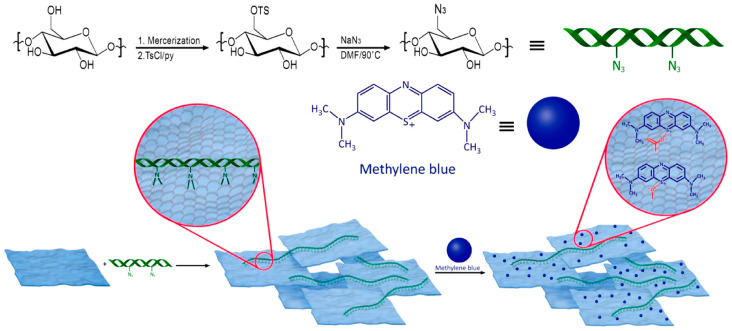
Schematic representation of the production procedure, as well as the exhibition of the carboxylate and phenoxide adsorption, GO sites of the hybrids towards methylene blue. Reproduced with permission from Reference [159]. Copyright ©2019, American Chemical Society.

Recently, Daniyal et al. have prepared hexadecyltrimethylammonium bromide (CTA) modified CNC/GO thin film and assessed its potential in sensing copper and nickel ions based on surface plasmon resonance (SPR) technique [157,158]. The authors initially prepared CTA-CNC solution and then 0.1 wt.% of GO was dispersed within the solution and sonicated at 70 °C for 1 h (Figure 8B). The obtained solution of CTA-CNC/GO was spin-coated and deposited as a thin layer on the glass substrate modified with a thin gold film. The authors demonstrated that the presence of CTA improved the sensitivity of the SPR. They revealed that the combination of SPR and CTA-CNC/GO has the potential to be employed as effective sensors, which can detect copper and nickel ions. In another research work, Beyranvand et al. produced hydrogel based on CNC/GO hybrid as a new adsorbent for methylene blue. During the preparation, azide-functionalized CNC was synthesized after CNC tosylation [159]. Then CNC-N_3_/GO was obtained via nitrene chemistry [160]. The production process of CNC-N_3_/GO, as well as its mechanism of action, is illustrated in Figure 9. It was demonstrated that the prepared hybrid was an excellent adsorbent of methylene blue owing to the higher adsorption capacity, reasonable contact time and recyclability. More recently, Zheng et al. have synthesized a modified CNC/GO hybrid as an efficient adsorbent of Dy (III). The authors used the evaporation-induced self-assembly (EISA) method to spontaneously form an imprinted film. Beforehand, they carried out an in situ selective oxidation of CNC using 2,2,3,3-tetramethylpiperidine-1-oxyl (TEMPO) for which C6 hydroxyl group was primarily oxidized to the carboxyl group (-COOH) [161]. It was found that such modification improved the stability of the TEMPO-modified CNC through strong electrostatic repulsion on one hand and on the other hand, it offered more surface active sites for the adsorption of Dy (III). The latter was further improved by the introduction of GO, which created extra bonding sites to Dy (III) and enhanced the adsorption capacity of TEMPO-CNC/GO hybrid. These authors reported that the developed green hybrid was efficient and had a strong regeneration performance.

**Figure 10:**
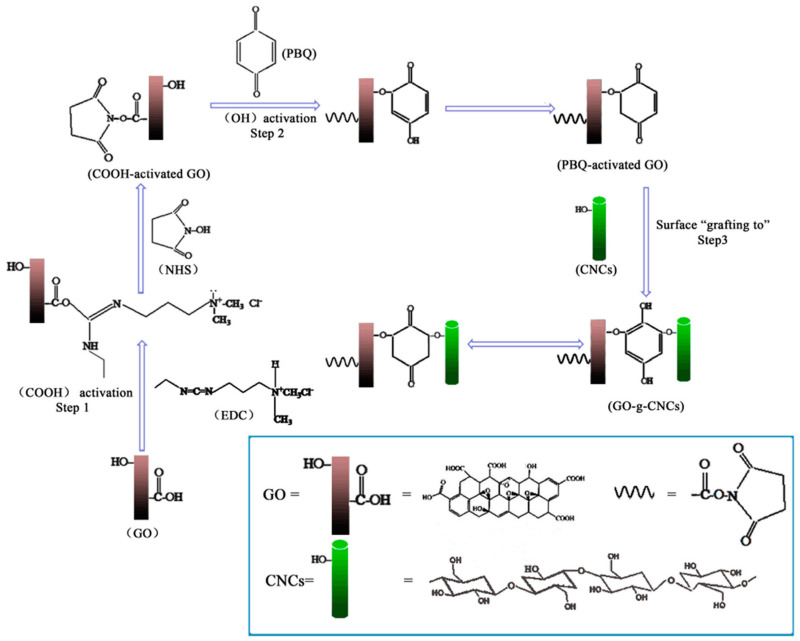
Schematic procedure of the preparation of the magnetic@GO-grafted-CNC@MIP. Reproduced with permission from Reference [163]. Copyright ©2017, Springer.

The preparation and design of molecularly imprinted polymers (MIPs) is a multidisciplinary field, which encompasses various aspects of molecular recognition, biomimetic biology and polymer chemistry. MIPs preparation involves arranging functional monomers around a template, followed by polymerization with the presence of cross-linkers and a suitable initiator through covalent, semi-covalent or non-covalent intermolecular interactions and finally template removal. Such an approach has been recently explored to produce CNC/GO-based composites as molecular imprinted electrochemical sensors, which exhibited outstanding features. For instance, Anirudhan et al. have prepared MIP of silylated GO and chemically modified CNC using a drop cast method for the selective sensing of cholesterol [162]. The authors incorporated ZnO to CNC to enhance their conductivity. The electrochemical studies were carried out using cyclic voltammetry and differential pulse voltammetry. This sensor achieved good stability and reproducibility, low detection limit and wide linear range. The optimum pH, equivalent to the blood pH, was 7.4 and the optimum response time was only 10 min. In another work, Wang et al. manufactured a CNC/GO-based MIP for the selective extraction and fat adsorption of synthetic antibiotics (fluoroquinolones, FQs), which can accumulate as residues in river water, causing a hazard for living organisms [163]. The preparation process of the magnetic@GO-grafted-CNC@MIP is depicted in Figure 10. It was found that the utilization of CNC and GO as substrates can improve the properties such as the stability, selectivity and affinity of MIPs compared to the conventional ones. The authors demonstrated that the prepared hybrid displayed an ultra-fast adsorption profile for FQs with high recognition and large detection limit range. They claimed that this method is accurate, effective, sensitive and simple, thereby appropriate for the detection of residual FQs in water sample.

**Figure 11:**
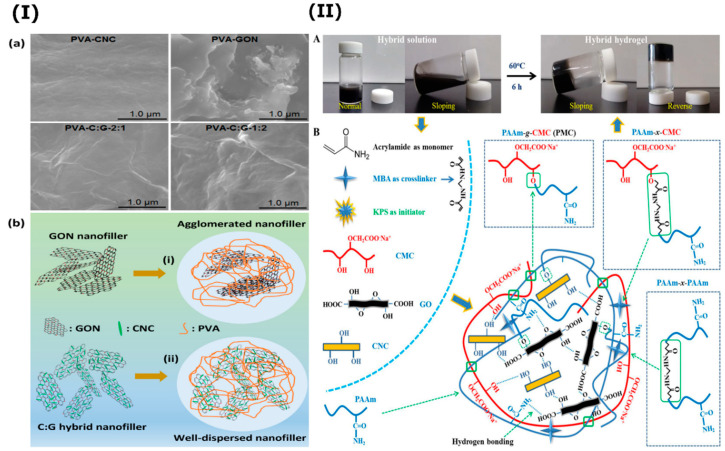
(**Ia**) Scanning electron microscopy (SEM) micrographs of PVA nanocomposites with CNC, GO and their hybrid (C: G-2:1 and C:G-1:2) and (**Ib**) schematic representations of the dispersion state of (i) GO and (ii) C: G hybrid within the PVA polymeric matrix. Reproduced with permission from Reference [171]. Copyright ©2016, Elsevier; (**II**) Schematic of the formation of the hydrogel: (**A**) Before and after heat treatment of PMC-GO1/CNC10.0 hybrid solution and (**B**) A suggested mechanism of physical and chemical interactions in the hybrid hydrogel system. Reproduced with permission from Reference [168]. Copyright ©2018, Elsevier.

The simultaneous incorporation of CNC and GO to numerous polymers such poly(3-hydroxybutyrate-co-3-hydroxy valerate) [164], poly-N-isopropyl acrylamide [165], poly(3,4-ethylenedioxythiophene) [166], poly(vinylidene fluoride) [167], polyacrylamide [168], poly(ε-caprolactone) [169], polylactic acid [170], poly(vinyl alcohol) (PVA) [171] and chitosan [172] was adopted as an efficient method to produce composites with excellent features since these nanofillers offer outstanding synergetic effects. Some surface modifications can be applied to CNC or GO to improve their dispersion and compatibility within the polymeric matrices. This type of nanocomposite found a wide range of applications in biosensing [165], plastic masks [172], tissue engineering [168,169], wastewater treatment [167], food packaging [173], supercapacitors [166], to cite a few. For instance, El Miri et al. evaluated the synergetic effect of CNC/GO as a functional hybrid to enhance the properties of PVA nanocomposites (Figure 11I). The nanocomposites were prepared via solvent casting method. The authors demonstrated that the tensile strength, toughness and Young’s modulus were respectively enhanced by 124%, 159% and 320% compared to the neat PVA. The strong interfacial interactions and the synergetic effect of 1D elongated CNC and 2D exfoliated GO, which improved the dispersion and avoided the agglomeration of the nanofillers, were also highlighted compared to the incorporation of pure CNC or GO. Such nanocomposite may find application in food packaging materials. In another recent study, Kumar et al. produced hybrid hydrogels containing polyacrylamide-sodium carboxymethylcellulose (PMC), GO and CNC via in situ free-radical polymerization (Figure 11II) [168]. The obtained composite displayed outstanding mechanical performance, self-healing behavior and shape-recovery feature. The authors claimed that such highly hydrated hybrid hydrogel with tailorable properties might provide a 3D microenvironment for tissue engineering applications.

### 4.3. CNC/RGO

The production of RGO, which is commonly performed by the exfoliation of pristine graphene followed by oxidation and reduction, may generate numerous defects such as grain boundaries, vacancies, Stone-Wales defects and macroscopic defects. These defects not only restrict its production at the industrial scale but also limit the full exploitation of its outstanding properties. Another obstacle in the practical use of RGO is the formation of irreversible agglomeration caused by the strong van der Waals interactions between graphene planes. Therefore, various attempts were made to reduce these drawbacks through RGO functionalization or incorporation of other additives such as vitamin C, green tea, protein bovine serum albumin and CNC, among others, to improve the properties and performance of the final derived nanocomposites and increase the number of its applications in several fields [28,173].

**Figure 12:**
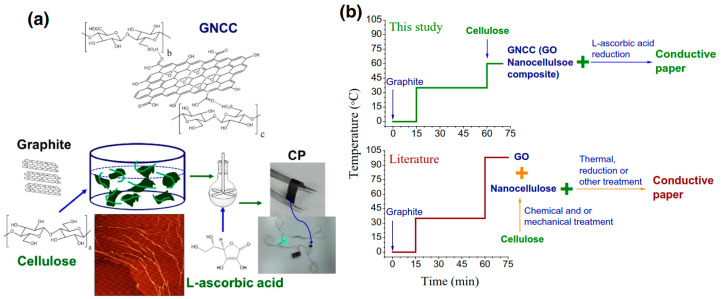
(**a**) A schematic flow diagram illustrates CNC/GO (GNCC) production using the one-pot method and further reduced to form conductive paper (CP, CNC/RGO). (**b**) A comparison of the present one-pot process with the conventional approach in literature. Reproduced with permission from Reference [177]. Copyright ©2019, Springer.

**Figure 13:**
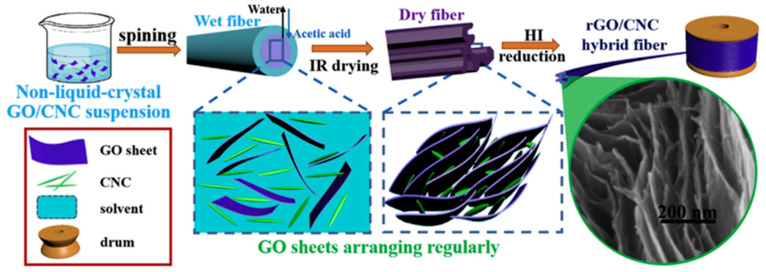
Schematic illustration of the preparation of CNC/RGO hybrid fiber. Reproduced with permission from Reference [130]. Copyright ©2018, Elsevier.

Nowadays, various unmodified CNC/RGO hybrids were actively explored for different applications such as sensors, flexible electronics, supercapacitors and photonic devices [174]. Several approaches used to produce some unmodified CNC/RGO hybrids have been reported for which the common method used to produce CNC/GO can be applied. Wan Khalid prepared COC/RGO nanocomposite by dispersion/ultrasonication of 1 mg RGO in ethanol and 1 mg of CNC in deionized water [175]. The supernatant was eliminated by centrifugation to recover the final hybrid. This latter, re-dispersed in ethanol, was drop-coated onto an electrode surface, which was intended to be used for electrochemical sensing of methyl paraben. The authors revealed that the obtained sensor exhibited good stability, reproducibility, selectivity toward methyl paraben and reusability compared to RGO-based sensor. Similarly, Nan et al. produced iridescent RGO/CNC film with advanced optical properties [176]. They prepared a suspension containing 1 wt.% of CNC and RGO floccules, which underwent an ultrasound treatment and dried using vacuum-assisted self-assembly (VASA) technique. The obtained films displayed regularly metallic iridescence owing to the homogeneous dispersion of RGO within the chiral nematic liquid crystals of CNC. The key factors to tune such behavior were the duration of ultrasonic treatment and the drying process. This iridescent hybrid exhibited better electrical properties in addition to the reversible change in color during the adsorption/desorption of water. The authors claimed that such a hybrid might find applications in photonic devices and biosensors. Recently, Wang et al. adopted a facile one-pot technique to prepare CNC/GO nanocomposite that was followed by a reduction using L-ascorbic acid to form CNC/RGO conductive paper [177]. The process, compared to the well-known ones, is schematically represented in Figure 12. Briefly, the exfoliation of graphite and the hydrolysis of cellulose occurred simultaneously in the reaction system, followed by subsequent reduction using green L-ascorbic acid. A conductive paper (CP) with high conductivity, excellent mechanical properties and thermal stability was then formed using ultrafiltration. It was stated that such CP can be used in implantable biosensors, smart textiles and portable micropower devices. In another research activity, Chen et al. proposed a new method to produce CNC/RGO hybrid, which was based on non-liquid-crystal spinning followed by a reduction using hydrogen iodide (HI), as schematized in Figure 13 [130]. The authors revealed that the incorporation of an alkaline media during the dispersion of CNC/RGO caused the electrostatic repulsion between CNC and GO sheets, leading to weaker hydrogen-bonding interaction and rendering the flowing process during spinning more homogeneous and easier. The authors demonstrated that the strength of RGO/CNC hybrid (230.6 MPa) was improved compared to pure RGO (157.5 Mpa). The hydrophilicity of the hybrid was also improved in addition to the high capacitive performance and conductivity. After that, such a hybrid was immersed in a polyvinyl alcohol acidic solution to fabricate flexible all-solid-state supercapacitor. The assembled supercapacitor achieved excellent bending stability, better flexibility, high energy density (5.1 mW h cm^−3^) and power density (496.4 mW cm^−3^). The authors claimed that the prepared hybrid easily meets the requirements of flexible or even wearable supercapacitor.

**Figure 14:**
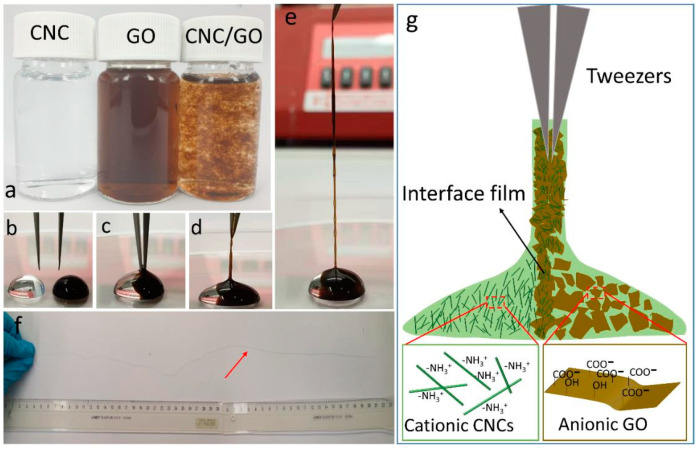
(**a**) suspensions of cationic CNC and GO and a dual precipitated complex after simple mixing; (**b**–**e**) CNC/GO hybrid filament drawing process; (**f**) a single dried CNC/GO hybrid filament with a diameter of ~33 µm and a length of 53 cm; (**g**) a scheme illustrating the CNC/GO hybrid filament drawing process. Reproduced with permission from Reference [178]. Licensed under a Creative Commons Attribution 3.0 International License (https://creativecommons.org/licenses/by/3.0/).

To further extend the number of applications, improve the different properties of CNC/RGO hybrids as well as their efficiency, numerous modifications of either CNC, RGO or both of them have been recently assessed. For instance, Zhao et al. produced electro-conductive nanocomposite based on CNC and TiO_2_-RGO. Firstly, GO prepared by the modified Hummers method was subjected to the photocatalytic reduction via TiO_2_. The obtained TiO_2_-RGO suspension was mixed with CNC suspension under ultrasonication. CNC/TiO_2_-RGO was then vacuum-filtered and dried. The obtained flexible transparent hybrid displayed improved electro-conductivity (9.3 S/m) with enhanced elastic modulus (3998 MPa) and tensile strength (18.1 MPa), stipulating that it can be used as a transparent flexible substrate for future electronic devices. In another work, Zhang et al. demonstrated the feasibility of the spinning of conductive filaments from oppositely charged nano-species, that is, cationic CNC and anionic RGO using interfacial nanoparticle complexation [178]. Initially, 2,3 dialdehyde cellulose was prepared by periodate oxidation, subjected to cationization with Girard’s reagent or aminoguanidine hydrochloride and passed through the double-chamber system of a microfluidizer to form cationic CNC. Droplets of aqueous suspensions (cationic CNC and anionic GN), placed adjacent to each other, generated continuous CNC/GO filaments, as shown in Figure 14. These latter were immersed into hydrogen iodide solution, washed and dried, to afford CNC/RGO hybrid filaments. These hybrids displayed an electrical conductivity of 3298 ± 167 S/m and tensile strength of 190.3 ± 8 MPa.

**Figure 15:**
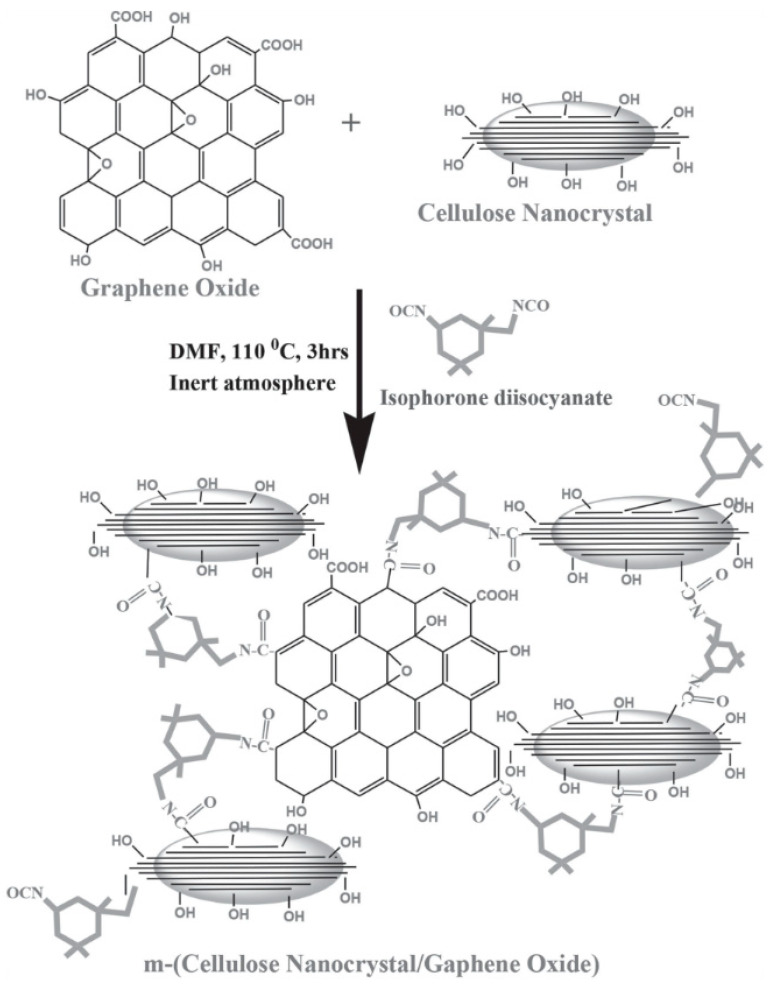
Reaction mechanism involved in modified CNC/GO synthesis. Reproduced with permission from Reference [181]. Copyright ©2015, Elsevier.

Kabiri and Namari described another interesting process for the preparation of CNC/RGO hybrid. They functionalized RGO with CNC via “click” coupling between terminated propargyl-functionalized CNC (PG-CNC) and azide-functionalized GO (GO-N_3_) [179]. After the surface azidation of GO, the “click” reaction between GO-N_3_ and PG-CNC, already synthesized by the Peng method [180], was performed using copper-catalyzed azide-alkyne cycloaddition. The reduction of the final nanocomposite dispersed in deionized water under sonication was carried out using hydrazine at 70 °C. The obtained free-dried CNC/RGO hybrid exhibited interesting physicochemical properties and thermal stability. In another study done by Sadasivyni et al., a transparent and eco-friendly CNC/RGO film for proximity sensing was developed [181]. The authors employed layer-by-layer spraying of modified CNC/GO nanocomposite, which was obtained as detailed in Figure 15, on lithographic patterns of interdigitated electrodes on polymer substrates. The modified nanocomposite was reduced using anhydrous hydrazine at 80 °C to generate a hydrophobic CNC/RGO hybrid. The obtained sensitive sensor allowed detecting a human finger interface within a distance of 6 mm with interesting response and recovery time interval. This sensor has potential to be used in various applications such as robotics, punching machines, smart phones, electronics and optoelectronics.

**Figure 16:**
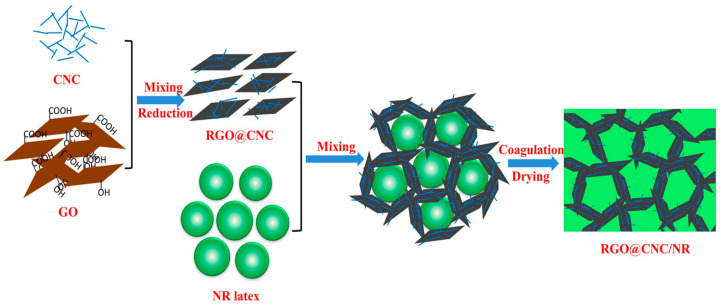
Schematic presentation of the synthesis of CNC/RGO/NR nanocomposite. Reproduced with permission from Reference [182]. Copyright ©2016, Elsevier.

On the other hand, several CNC/RGO-based polymer composites have been produced and assessed in several applications such as sensors, scaffolds in tissue engineering, food and drug packaging. The polymeric matrices tested include polyvinylidene chloride (PVDC) [173], natural rubber (NR) [182], polyethylene oxide [183], poly-lactic acid (PLA) [184,185] and polyamide 6 [186]. It was demonstrated that the incorporation of CNC/RGO conferred to the polymeric nanocomposites outstanding mechanical and thermal properties, interesting barrier features, low toxicity, high conductivity and so forth [183,184,186]. For instance, Cao et al. prepared a 3D interconnected CNC/RGO/NR network using a latex assembly method for which NR latex was incorporated into a CNC/RGO suspension [182]. The solid formed through the co-coagulation induced by an acidic solution was vacuum filtered. A schematic illustration of the process is provided in Figure 16. The obtained conductive structure displayed higher electric conductivity and better mechanical features with superior resistivity responses for organic liquids. Such nanocomposite can find application in sensing to discriminate various solvents leakage in chemical industries and environmental monitoring.

**Figure 17:**
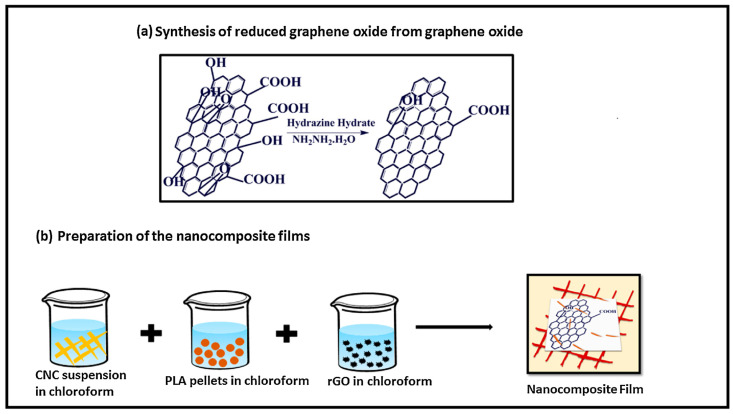
Schematic illustration of (**a**) the synthesis of reduced graphene oxide (RGO) from GO, (**b**) the method employed to prepare nanocomposite films. Reproduced with permission from Reference [185]. Copyright ©2017, Elsevier.

**Figure 18:**
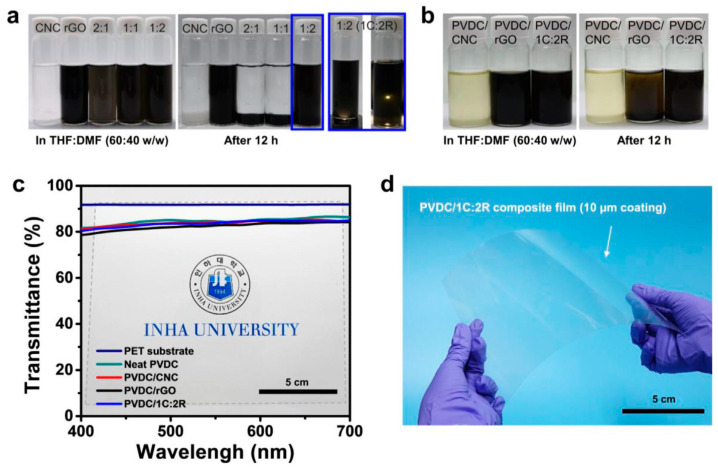
Dispersion stability of nanofillers. CNC, RGO and 1CNC:2RGO (1C:2R) hybrid in (**a**) THF:DMF co-solvent and (**b**) PVDC nanocomposite solutions of 0.1 wt.% fillers loading to PVDC. (**c**) Transmittance results of 0.1 wt.% PVDC/CNC, RGO and 1C:2R nanocomposite films. The 10 mm thick nanocomposite films were deposited on 125 mm thick PET substrates. (**d**) Large area (17 cm × 21 cm) PVDC/1C:2R–0.1 wt% nanocomposite film was obtained. Reproduced with permission from Reference [173]. Copyright ©2020, Elsevier.

In other research, Pal et al. assessed the combined effect of CNC and RGO in PLA nanocomposite as a scaffold in tissue engineering [185]. They initially prepared CNC and RGO via acid hydrolysis and modified Hummer’s method, respectively and then employed a solution casting approach to produce CNC/RGO/PLA hybrid. The detailed preparation procedure is schematized in Figure 17. Compared to pristine PLA, the developed hydrophilic hybrid film revealed higher thermal stability, significantly increased tensile strength up to 23% with and enhancement in elongation at break, showing its ductile behavior. Moreover, the antibacterial activity against both Gram-negative *Escherichia coli* (*E. coli*) and Gram-positive *Staphylococcus aureus* (*S. aureus*) bacterial strains was highlighted. The *in-vitro* cytotoxicity assay indicated the non-toxicity of the nanocomposite film toward fibroblast cell line (NIH-3T3) as well. More recently, interesting research has been conducted by You et al. [173], who introduced the hybrid CNC/RGO to the solution of PVDC (Figure 18). The precipitated sample was vacuum dried to produce the CNCN/RGO/PVDC nanocomposite. The transparency of the CNC/RGO/PVDC coated on PET substrate was determined as 84% at 550 nm wavelength by UV–visible spectrometer in the regular transmission mode (Figure 18). It was proved that the utilization of stable dispersion of CNC/RGO enabled the fabrication of optically clear and thermostable nanohybrid film with improved barrier characteristics against water and oxygen. The authors claimed that the developed approach to produce CNCN/RGO/PVDC nanocomposite was effective and the obtained hybrid film is considered a potential candidate for food and drug packaging.

## 5. Summary and Outlook

During the last decade, significant advances have been made in the preparation, characterization and application of CNC/GNM hybrids. This article is a brief review of this fast-growing research area and intended to highlight the up-to-date studies and utilization of CNC/GNM hybrids. Firstly, we have introduced some basic concepts of nanocellulose and GNM, then summarized their preparation methods and properties, with a particular focus on their outstanding features to elucidate their unique attributes. The different preparation processes of CNC/GNM have been discussed as well as their properties. Furthermore, to well understand the characteristics of theses hybrids, their different applications have been provided.

CNC/GNM hybrid-based materials displayed interesting innovative features due to synergetic effects, which are unachievable by taking CNC and GNM materials separately. It is shown that the combination of the diversity and specificity of both CNC and GNM not only expands the number of applications but also has indisputable advantages to benefit their unique attributes. These hybrids hold a cornucopia of favorable properties that warrant their employment in the fields of sensing, catalysis, separation, electronics, optics, biomedical, energy storage, to name a few. Nonetheless, the development of CNC/GNM hybrid-based materials is relatively a new concept, which is mostly limited to academic discipline but is expected that CNC/GNM hybrids will certainly be commercially available in the future, which will attract more research attention not only in various applications but also to achieve multifunctional multi-systems and open new perspectives. Moreover, the practical application of such hybrids as next-generation materials requires further improvements in functionality and performance in addition to the reduction of the production costs and the environmental impacts.
